# Supplementary material for: Cyanobacterial Blooms and the Presence of Cyanotoxins in the Brazilian Amazon
Source: Toxins (Basel). 2025 Jun 11;17(6):296. doi: 10.3390/toxins17060296 (PMC12197653; doi:10.3390/toxins17060296)
Supplement: Supplementary file 1 [file toxins-17-00296-s001.zip › toxins-3517353-supplementary.pdf]

Supplementary Materials

Review

Cyanobacterial Blooms and the Presence of Cyanotoxins in the Brazilian Amazon

Maria Paula Cruz Schneider [1,*], Elane Cunha [2], Lucas Silva [1], James Leão [3], Vanessa Costa Tavares [4], Eliane Brabo de Sousa [4] and Silvia Faustino [5]

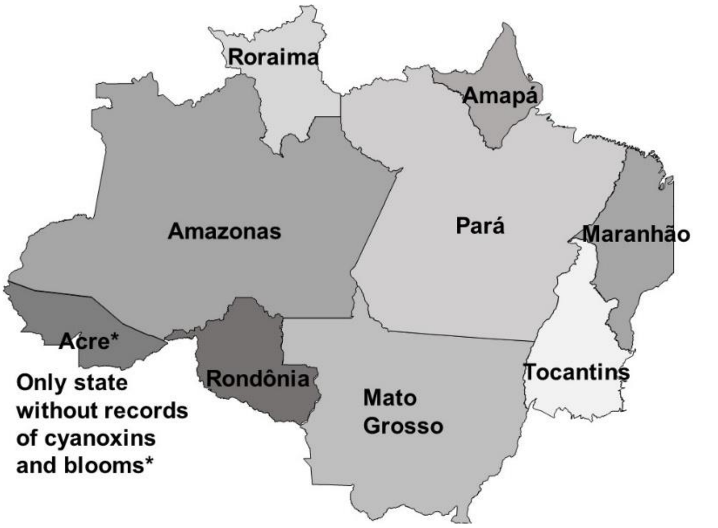

Figure S1. States of the Brazilian Legal Amazon.

Table S1. Studies with the main species of cyanobacteria and toxins detected for each state of the Brazilian Legal Amazon. CYN: cilindrospermopsin; MC: total microcistin; MC-LR: microcistin-LR; STX: saxitoxin.

Brazilian Legal Amazon states	Authors	Bloom-associated cyanobacteria	Detected toxins
Acre<br>(Only region without records)	-	-	-
Amapá<br>(5 publications)	**2 Master's Dissertations**<br>Dias, 2007 [12]<br>Barbosa, 2015 [13]<br>**1 PhD Thesis**<br>Oliveira, 2018 [14]<br>**2 Articles**<br>Oliveira et al., 2019 [15]<br>Cavalcante et al., 2021 [16]	*Aphanizomenon* sp.<br>*Anabaena* sp.<br>*Dolichospermum planctonicum,*<br>*Dolichospermum spiroides*<br>*Geitlerinema amphibium*<br>*Leptolyngbya boryana*<br>*Limnothrix planctonica*<br>*Microcystis aeruginosa*<br>*Phormidium* sp.<br>*Planktothrix agardhii*<br>*Pseudanabaena* sp.<br>*Radiocystis fernandoi*<br>*Raphidiopsis* sp.	MC-LR<br>MC

Amazonas<br>(3 publications)	**3 articles**<br>Pascoaloto, Soares e Gomes, 2021 [17],<br>Arcos et al. 2018 [18]<br>Melo et al., 2024 [19]	*Dolichospermum planctonicum*,<br>*Planktotrix agardhii*<br>*Microcystis aeruginosa*<br>*Microcystis wesenbergii*	No toxins
Maranhão<br>(1 publication)	**1 article**<br>Cutrim et al., 2019 [20]	*Microcystis aeruginosa*<br>*Oscillatoria*	No toxins
Mato Grosso<br>(1 publication)	**1 article**<br>Da Costa et al., 2017 [21]	*Raphidiopsis raciborskii*<br>*Microcystis aeruginosa*<br>*Planktothrix* spp.	No toxins
Pará<br>(26 publications)	**14 articles**<br>Schmidt, 1982 [22]<br>Vieira et al., 2005 [23]<br>Sá et al., 2010 [24]<br>Brandão et al., 2017 [25]<br>Lobo et al., 2017 [26]<br>Lobo et al., 2018 [27]<br>Kraus et al., 2019 [28]<br>Silva et al., 2019 [29]<br>Torres et al., 2020 [30]<br>Gomes et al. 2021 [31]<br>Lopes et al., 2024 [32]<br>Maciel et al., 2024 [33]<br>Silva et al., 2024 [34]<br>Leal et al., 1999 [35]<br>**2 Books**<br>Braun, 1952 [36]<br>Sioli, H. 1984 [37]<br>**2 PhD Thesis**<br>Vieira, J. M, dos S., 2002 [38]<br>Silva, S.C.F da, 2020 [39]<br>**6 Master's Dissertations**<br>Alves, C. P. P., 2011 [40]<br>Sousa, E.B. 2017 [41]<br>Silva, S.C.F da, 2012 [42]<br>Araújo, J. L.de, 2020 [43]<br>Oliveira, F. A, 2022 [44]<br>Souza, D. A. 2015 [45]<br>**3 technical reports**<br>IEC, 2024 [46]	*Anabaena* spp.<br>*Anabaena variablilis*<br>*Dolichospermum* spp.<br>*Dolichospermum circinalis*<br>*Dolichospermum planctonicum*<br>*Dolichospermum solitarium*<br>*Dolichospermum spiroides*<br>*Dolichosphermum circinale*<br>*Lyngbya* spp.<br>*Microcystis aeruginosa*<br>*Microcystis panniformis*<br>*Microcystis protocystis*<br>*Microcystis* spp.<br>*Microcystis wesenbergii*<br>*Nostoc* sp.<br>*Phormidium* sp.<br>*Planktothrix agardhii*<br>*Planktothrix isothrix*<br>*Planktothrix* sp.<br>*Pseudanabaena mucicola*<br>*Radiocystis fernandoii*<br>*Raphidiopsis curvata*<br>*Synechocystis aquatilis*<br>*Synechocystis* sp.	MC-LR<br>MC<br>STX

	Rebelo, Lima e Silva, 2003 [47]<br>Oliveira et al. 2025 [48]		
Rondônia<br>(5 publications)	**1 article**<br>Pinheiro et al., 2023 [49]<br>**1 PhD Thesis**<br>Nascimento, 2012 [50]<br>**3 Master's Dissertations**<br>Hurtado, 2014 [51]<br>Bandeira, 2021 [52]<br>Kozerski, 2023 [53]	*Microcystis* sp.<br>*Microcystis aeruginosa,*<br>*M. flos-aquae*<br>*Microcystis panniformes*<br>*Oscillatoria* sp.<br>*Planktothrix* sp.<br>*Planktothrix agardhii*<br>*Planktolyngbya limnetica*<br>*Raphidiopsis raciborskii*	MC
Roraima<br>(1 publication)	**1 Dissertation**<br>Furtado, 2011 [54]	*Planktothrix agardhii*	Unspecified toxin with hepatotoxic effect
Tocantins<br>(5 publications)	**3 articles**<br>Silva et al., 2003 [55]<br>Silva et al., 2005 [56]<br>**2 PhD Theses**<br>Pereira, 2002 [57]<br>Silva, 2014 [58]<br>**1 Dissertation**<br>Silva, 2009 [59]	*Microcystis aeruginosa*<br>*Planktolyngbya limnetica*<br>*Radiocystis fernandoi*<br>*Raphidiopsis raciborskii*	MC<br>STX<br>CYN

Table S2. c

Orders	Species
Chroococcales	1. *Aphanocapsa annulata*
	2. *Aphanocapsa elachista*
	3. *Aphanocapsa holsatica*
	4. *Aphanocapsa incerta*
	5. *Aphanocapsa stagnalis,*
	6. *Aphanocapsa biformis*
	7. *Aphanocapsa delicatissima*
	8. *Aphanocapsa holsatica*
	9. *Aphanocapsa koordesi*
	10. *Aphanocapsa montana*
	11. *Aphanocapsa parasitica*
	12. *Aphanocapsa* spp.
	13. *Aphanothece* spp.
	14. *Chroococcus dispersus*
	15. *Chroococcus limneticum*
	16. *Chroococcus minor*
	17. *Chroococcus minutus*

	18. *Chroococcus sonorensis*
	19. *Chroococcus* sp.
	20. *Chroococcus turgidus*
	21. *Coelomorom* sp.
	22. *Coelomoron microcystoides,*
	23. *Coelomoron pusillum*
	24. *Coelomoron tropicale*
	25. *Coelomoron tropicalis*
	26. *Coelosphaerium kuetzingianum*
	27. *Coelosphaerium* sp.
	28. *Dactylococcopsis pectinatellophila*
	29. *Dactylococcopsis rhaphidioides*
	30. *Limnococcus limneticus*
	31. *Merismopedia tenuissima*
	32. *Merismopedia viridis*
	33. *Merismopedia elegans*
	34. *Merismopedia glauca*
	35. *Merismopedia minima*
	36. *Merismopedia punctata*
	37. *Merismopedia* spp.
	38. *Merismopedia trolleri*
	39. *Microcrocis pulchella*
	40. *Microcystis aeruginosa*
	41. *Microcystis botrys*
	42. *Microcystis novacekii*
	43. *Microcystis panniformis*
	44. *Microcystis protocystis*
	45. *Microcystis pseudofilamentosa*
	46. *Microcystis robusta*
	47. *Microcystis* spp.
	48. *Microcystis viridis*
	49. *Microcystis wesenbergii*
	50. *Radiocystis fernandoi*
	51. *Snowella fennica*
	52. *Snowella lacustris,*
	53. *Snowella litoralis*
	54. *Sphaerocavum brasiliense*
	55. *Sphaerocavum* sp.
	56. *Synechocystis aquatilis*
	57. *Woronichinia naegeliana*
	58. *Woronichinia* sp.
Chroococcidiopsidales	1. *Chlorogloea microcystoides*
	2. *Gloeocapsa* sp.

	3. *Gloeocapsarupestris* sp.
Geitlerinematales	1. *Geitlerinema splendidum,*<br>2. *Geitlerinema amphibium,*<br>3. *Geitlerinema* sp.
Gomontiellales	1. *Borzia brevis*<br>2. *Borzia* sp.<br>3. *Komvophoron* sp.
Leptolyngbyales	1. *Alkalinema pantanalense*<br>2. *Leptolyngbya* cf. *tenuissima*<br>3. *Leptolyngbya* sp.<br>4. *Leptolyngbya tenuis*<br>5. *Planktolyngbya limnetica*<br>6. *Planktolyngbya* sp.<br>7. *Scytolyngbya* sp.
Nodosilineales	1. *Epigloeosphaera brasilica*<br>2. *Rhabdoderma lineare*<br>3. *Rhabdoderma elipsoidea.*<br>4. *Rhabdoderma* spp<br>5. *Romeria* sp.
Nostocales	1. *Anabaena circinalis*<br>2. *Anabaena fertilissima*<br>3. *Anabaena flos-aquae*<br>4. *Anabaena planctonica*<br>5. *Anabaena* spp.<br>6. *Aphanizomenon anabaena*<br>7. *Aphanizomenon flos aquae*<br>8. *Aphanizomenon gracile*<br>9. *Aphanizomenon* sp.<br>10. *Aphanizomenon tropicalis*<br>11. *Cilindrospermopsis raciborskii (currently Raphidiopsis raciborskii)*<br>12. *Dolichospermum planctonicum*<br>13. *Dolichospermum circinale*<br>14. *Dolichospermum crassum*<br>15. *Dolichospermum flosaquae*<br>16. *Dolichospermum planctonicum*<br>17. *Dolichospermum sigmoideum*<br>18. *Dolichospermum solitarium*<br>19. *Dolichospermum* spp.<br>20. *Dolichospermum spiroides*<br>21. *Cuspidothrix* sp.<br>22. *Hapalosiphon* sp.<br>23. *Raphidiopsis curvata*

	24. *Raphidiopsis mediterranea*<br>25. *Raphidiopsis* sp.<br>26. *Nostoc calcicola*<br>27. *Nostoc* cf. *muscorum*<br>28. *Nostoc* sp.<br>29. *Nostochopsis* sp.
Oscillatoriales	1. *Arthrospira jenneri*<br>2. *Arthrospira skujae*<br>3. *Blennothrix* sp.<br>4. *Cephalothrix lacustris*<br>5. *Cephalothrix* sp.<br>6. *Lyngbya* spp.<br>7. *Oscillatoria americana*<br>8. *Oscillatoria jenensis*<br>9. *Oscillatoria limosa*<br>10. *Oscillatoria obtusa*<br>11. *Oscillatoria perornata*<br>12. *Oscillatoria princeps*<br>13. *Oscillatoria refringens*<br>14. *Oscillatoria rubencens*<br>15. *Oscillatoria sancta*<br>16. *Oscillatoria* spp.<br>17. *Planktothrix isothrix*<br>18. *Phormidium formosum*<br>19. *Phormidium tergestinum*<br>20. *Phormidium* spp.<br>21. *Planktothrix agardhii*<br>22. *Planktothrix mougeotii*<br>23. *Planktothrix* spp.<br>24. *Trichodesmium* sp.<br>25. *Tychonema bourellyi*
Pleurocapsales	1. *Hydrococcus cesatii*<br>2. *Hydrococcus rivularis*
Pseudanabaenales	1. *Limnothrix planctonica*<br>2. *Limnothrix redekei*<br>3. *Pseudanabaena catenata*<br>4. *Pseudanabaena mucicola,*<br>5. *Pseudanabaena galeata*<br>6. *Pseudanabaena limnetica*
Spirulinales	1. *Spirulina* sp.
Synechococcales	1. *Anathece* cf. *minutissima,*<br>2. *Anathece* sp.

	3. *Jaaginema neglecta*
	4. *Jaaginema pseudogeminatum*
	5. *Synechococcus elongata*
	6. *Synechococcus* spp.
